# Supplementary material for: DMRG/FQ: A Polarizable Embedding Approach Combining Density Matrix Renormalization Group and Fluctuating Charges
Source: J Chem Theory Comput. 2026 Feb 10;22(4):1811–22. doi: 10.1021/acs.jctc.5c02116 (PMC12937113; doi:10.1021/acs.jctc.5c02116)
Supplement: Supplementary file 1 [file ct5c02116_si_001.pdf]

Supporting Information for: DMRG/FQ: a Polarizable Embedding Approach Combining Density Matrix Renormalization Group and Fluctuating Charges

Matteo Rinaldi,† Chiara Sepali,† Alicia M. Kirk,† Claudio Amovilli,‡ and Chiara Cappelli*,†

†*Scuola Normale Superiore, Piazza dei Cavalieri 7, I-56126 Pisa, Italy*

‡*Dipartimento di Chimica e Chimica Industriale, Università di Pisa, via Moruzzi 13, I-56124 Pisa, Italy*

E-mail: chiara.cappelli@sns.it

Contents

S1 Molecular dynamics of DCBT in acetonitrile **S3**

S2 Benchmarking of DMRG/FQ excitation energies **S5**

S3 Statistical summary of the distributions of calculated vertical excitation energies **S8**

S4 Convergence of vertical excitation energies with respect to the number of snapshots extracted from the MD simulation **S9**

S5 Excitation energies and solvatochromic shifts **S10**

S6 Digitized DCBT absorption spectra from the literature **S11**

S1 Molecular dynamics of DCBT in acetonitrile

Molecular dynamics simulations were performed using GROMACS 2020.4.[1] Parameters for DCBT were generated with ACPYPE-*antechamber*[2,3] using the General Amber Force Field (GAFF),[4] AM1-BCC charges,[5] and molecular geometry optimised prior using Gaussian16 at the MP2/6-31G(d) level of theory with implicit acetonitrile incorporated using the Polarizable Continuum Model (PCM).[6–9] Parameters for a six-point gaff-derived acetonitrile model were sourced from the work of Kowsari and co-workers.[10] DCBT was solvated with approximately 17000 acetonitrile molecules in a cubic box of 11.54 nm. The system was minimised using steepest descent. Short-range electrostatic and Van de Waals cut-offs were set to 1.2 nm and long range electrostatic interactions were treated using particle-mesh Ewald (PME) with periodic boundary conditions.[11] Throughout all stages, strong position restraints (10000 kJ/mol/nm^2) were applied to DCBT to maintain the planarity of the conjugated core while the butyl side chains were free to move. Using an NVT ensemble, the system was heated and equilibrated to 298.15 K for 1 ns using the velocity-rescaling method (0.1 ps coupling constant) and time step of 2 fs. All bonds were constrained using the LINCS algorithm.[12,13] The system density was then equilibrated for 2ns under the NPT ensemble using the Berendsen barostat (2 ps coupling constant). Returning to the NVT ensemble, a final production run was performed for 30 ns from which 200 uncorrelated snapshots were extracted at regular 150 ps intervals. The MDanalysis program was used to calculate the radical distribution function of the solvent (Figure S1).[14]

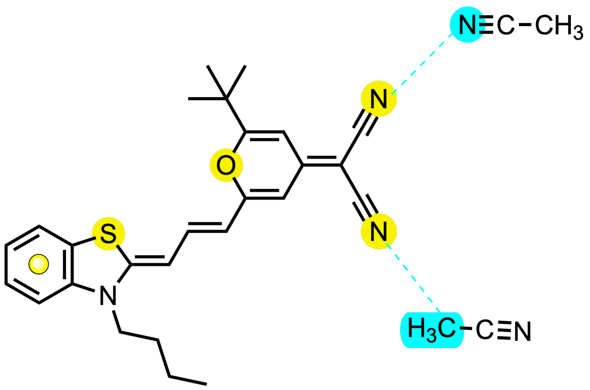

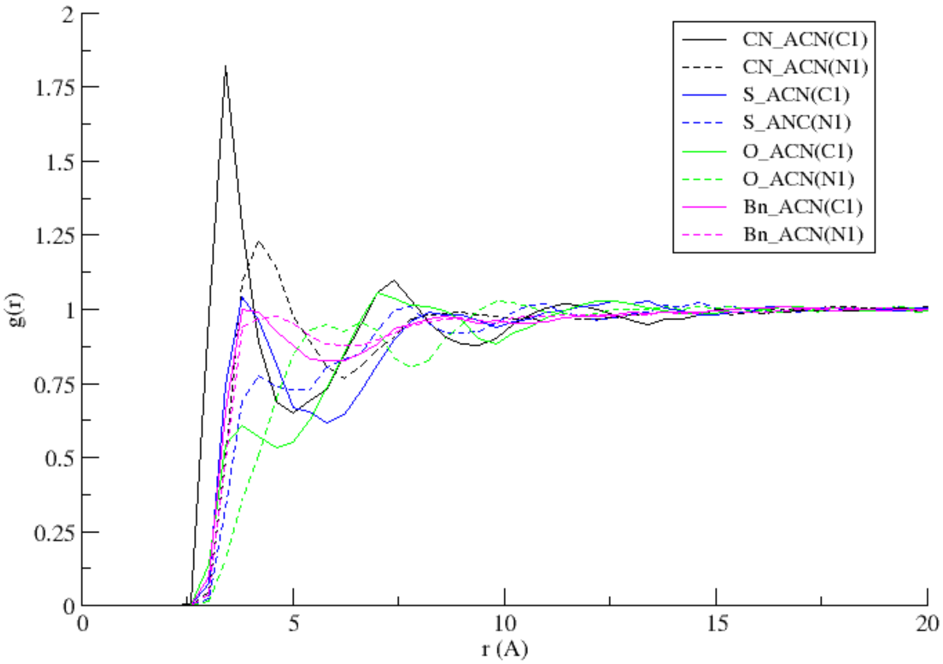

Figure S1: Radial distribution function (RDF) for selected DCBT and acetonitrile (ACN) atom pairs. Top diagram highlights in yellow DCBT atoms from which the RDF is measured; note Bn refers to the centre point of the benzene moiety; highlighted in blue are ACN(C1), which refers to the acetonitrile methyl carbon and ACN(N1), which refers to the acetonitrile nitrogen. Ordering of ACN methyl groups toward DBCT nitrile substituents (black lines) is evident.

S2 Benchmarking of DMRG/FQ excitation energies

Table S1: Computed $n \rightarrow \pi^*$ vertical excitation energies of acetone with two water molecules hydrogen-bonded to the carbonyl oxygen (single structure), for a range of basis sets, active spaces, and solvation models. For the (4,3) and (6,5) active spaces, "GS (2,2)" denotes the use of the (2,2) active space for the GS and the (4,3) active space for the ES, while "GS (4,4)" denotes the (4,4) active space for the GS and the (6,5) active space for the ES.

Active space	M	Basis set	Solvent model							
			Gas-phase		ESPF		FQ[a]		FQ[b]	
				GS(2,2)		GS(2,2)		GS(2,2)		GS(2,2)
(4,3)	100	6-31g*	4.57	4.52	4.60	4.55	4.69	4.64	4.92	4.87
		cc-pVDZ	4.58	4.53	4.61	4.57	4.70	4.65	4.92	4.88
		aug-cc-pVDZ	4.58	4.53	4.63	4.59	4.73	4.69	4.99	4.95
				GS(4,4)		GS(4,4)		GS(4,4)		GS(4,4)
(6,5)	100	6-31g*	5.01	4.94	5.06	5.00	5.15	5.08	5.39	5.32
		cc-pVDZ	5.03	4.96	5.08	5.02	5.16	5.09	5.47	5.40
		aug-cc-pVDZ	5.03	4.97	5.11	5.04	5.20	5.14	5.47	5.41
(12,10)	200	6-31g*	4.97	–	5.08	–	5.17	–	5.37	–
		cc-pVDZ	4.99	–	5.10	–	5.18	–	5.38	–
		aug-cc-pVDZ	5.01	–	5.15	–	5.25	–	5.49	–
(24,22)	300	6-31g*	4.83	–	4.85	–	4.94	–	5.13	–
		cc-pVDZ	4.84	–	4.87	–	4.95	–	5.13	–
		aug-cc-pVDZ	4.84	–	4.89	–	4.99	–	5.20	–

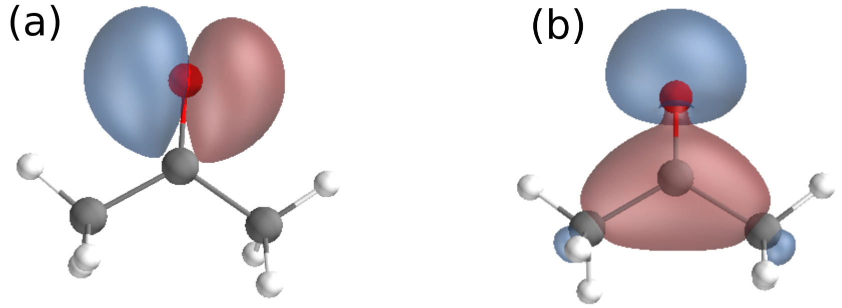

Figure S2: **a)** 'Antisymmetric' n orbital included in the (4,3) and (6,5) active spaces. **b)** 'Symmetric' orbital of n character, replacing the 'antisymmetric' one in the (4,3) and (6,5) active spaces after GS optimization.

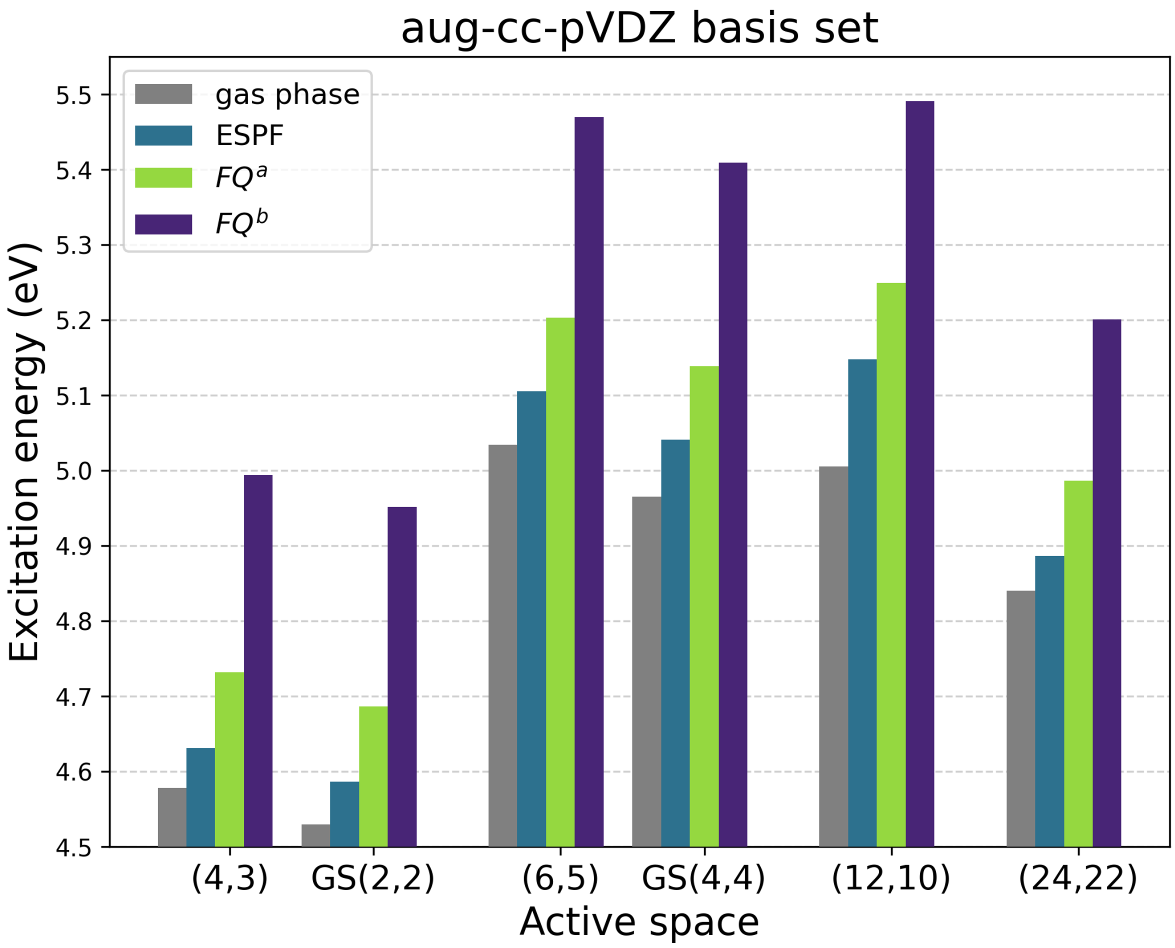

Figure S3: Computed $n \rightarrow \pi^*$ vertical excitation energies (eV) of acetone hydrogen bonded to two water molecules (single structure) using the aug-cc-pVDZ basis set. Results are compared for selected, active spaces and solvation models. "GS (2,2)" denotes the use of the (2,2) active space for the GS and the (4,3) active space for the ES, while "GS (4,4)" denotes the (4,4) active space for the GS and the (6,5) active space for the ES.

S3 Statistical summary of the distributions of calculated vertical excitation energies

Table S2: Statistical summary of the distribution of $n \to \pi^*$ vertical transition energies (eV) calculated for acetone across the different snapshots, including mean, mode, median and standard error of the mean (SEM). Results are compared for the selected active spaces (CASSCF (12,10) and DMRG (24,22)) and solvation models.

Active space	Solvent model	Mean (eV)	Mode (eV)	Median (eV)	SEM (eV)
(12,10)	FQ^a	5.30	5.31	5.24	0.02
(12,10)	FQ^b	5.69	5.75	5.74	0.02
(24,22)	ESPF	4.96	5.01	4.96	0.01
(24,22)	FQ^a	4.89	4.90	4.89	0.01
(24,22)	FQ^b	5.06	4.92	5.07	0.01

Table S3: Statistical summary of the distribution of $\pi \to \pi^*$ vertical transition energies (eV) calculated for DCBT across the different snapshots, including mean, mode, median, and standard error of the mean (SEM). Results are compared for the selected active spaces (CASSCF (8,8) and DMRG (30,27)) and the FQ^b solvent model.

Active space	Solvent model	Mean (eV)	Mode (eV)	Median (eV)	SEM (eV)
(8,8)	FQ^b	3.50	3.43, 3.60, 3.63, 3.68	3.48	0.02
(30,27)	FQ^b	3.32	3.36	3.30	0.02

S4 Convergence of vertical excitation energies with respect to the number of snapshots extracted from the MD simulation

Table S4: Mean excitation energy of the $n \rightarrow \pi^*$ transition (eV) of acetone, with standard error and 95% confidence interval, evaluated over 50, 100, 150, and 200 snapshots.

Level of theory	Number of snapshots	Mean (eV)	SEM (eV)	95% (eV) C.I.
CASSCF(12,10)/ FQ^a	50	5.679	0.050	0.098
	100	5.704	0.035	0.068
	150	5.711	0.027	0.054
	200	5.688	0.024	0.048
CASSCF(12,10)/ FQ^b	50	5.254	0.043	0.085
	100	5.307	0.030	0.059
	150	5.303	0.024	0.047
	200	5.302	0.020	0.040
DMRG(24,22)/ESPF	50	4.923	0.026	0.051
	100	4.953	0.017	0.033
	150	4.954	0.013	0.026
	200	4.957	0.011	0.021
DMRG(24,22)/FQ^a	50	4.861	0.025	0.050
	100	4.892	0.018	0.035
	150	4.888	0.014	0.027
	200	4.890	0.011	0.022
DMRG(24,22)/FQ^b	50	5.051	0.030	0.060
	100	5.074	0.021	0.041
	150	5.067	0.016	0.032
	200	5.064	0.014	0.028

Table S5: Mean excitation energy of the $\pi \rightarrow \pi^*$ transition (eV) of DCBT, with standard error and 95% confidence interval, evaluated over 50, 100, 150, and 200 snapshots.

Level of theory	Number of snapshots	Mean (eV)	SEM (eV)	95% C.I. (eV)
CASSCF(8,8)/FQ^b	50	3.544	0.039	0.077
	100	3.523	0.026	0.052
	150	3.499	0.021	0.040
	200	3.497	0.018	0.035
DMRG(30,27)/FQ^b	50	3.374	0.036	0.071
	100	3.351	0.024	0.047
	150	3.325	0.019	0.037
	200	3.322	0.016	0.032

S5 Excitation energies and solvatochromic shifts

Table S6: Excitation energies (ε) and water-to-vacuum solvatochromic shifts ($\delta = \omega_{\text{solv}} - \omega_{\text{gas}}$) of the $n \rightarrow \pi^*$ transition of acetone in aqueous solution, computed at the DMRG(24,22)[300]/aug-cc-pVDZ and CASSCF(12,10)/aug-cc-pVDZ levels of theory using different solvent models (ESPF, FQ$^{(a,b)}$), together with experimental values.

Active space	Solvent model	ε (eV)	δ (eV)
(12,10)	gas phase	5.01	–
(12,10)	FQ^a	5.30	0.29
(12,10)	FQ^b	5.69	0.68
(24,22)	gas phase	4.84	–
(24,22)	ESPF	4.96	0.12
(24,22)	FQ^a	4.89	0.05
(24,22)	FQ^b	5.06	0.22
Exp.	gas phase	4.46,[15] 4.48[16]	–
Exp.	water	4.68,[15] 4.69[16]	0.22,[15] 0.21[16]

Table S7: Excitation energies (ε) and solvatochromic shifts ($\delta = \omega_{\mathrm{solv}} - \omega_{\mathrm{gas}}$) of the $\pi \rightarrow \pi^*$ transition of DCBT in acetonitrile, computed at the DMRG(30,27)/FQ^b[300]/6-31G* and CASSCF(8,8)/FQ^b/6-31G*level, together with digitized TD-DFT/IEFPCM/def2-TZVP, SA(2)-CASSCF/C-PCM(6,5)/6-31G* literature data and experimental values.

Level of theory	Solvent model	ε (eV)	δ (eV)
TD-DFT/IEFPCM	MCH	2.07	–
TD-DFT/IEFPCM	ACN	2.45	-0.38
SA(2)-CASSCF(6,5)	gas-phase	4.44	–
SA(2)-CASSCF/C-PCM(6,5)	DMSO	3.68	-0.76
CASSCF(8,8)	gas phase	3.94	–
CASSCF(8,8)	crystal structure	3.91	–
CASSCF(8,8)/FQ^b	ACN	3.50	-0.44,-0.41
DMRG(30,27)	gas phase	3.73	–
DMRG(30,27)	crystal structure	3.66	–
DMRG(30,27)/FQ^b	ACN	3.32	-0.41,-0.34
Exp.	MCH	2.12	–
Exp.	ACN	2.27	-0.15

S6 Digitized DCBT absorption spectra from the literature

Data from references 17 and 18 were extracted using the WebPlotDigitizer software.[19] In particular, from Figure 2 of ref. 17, we selected the left inflection point of each relevant band to reduce the error arising from the neglect of nuclear structure effects in the transition.

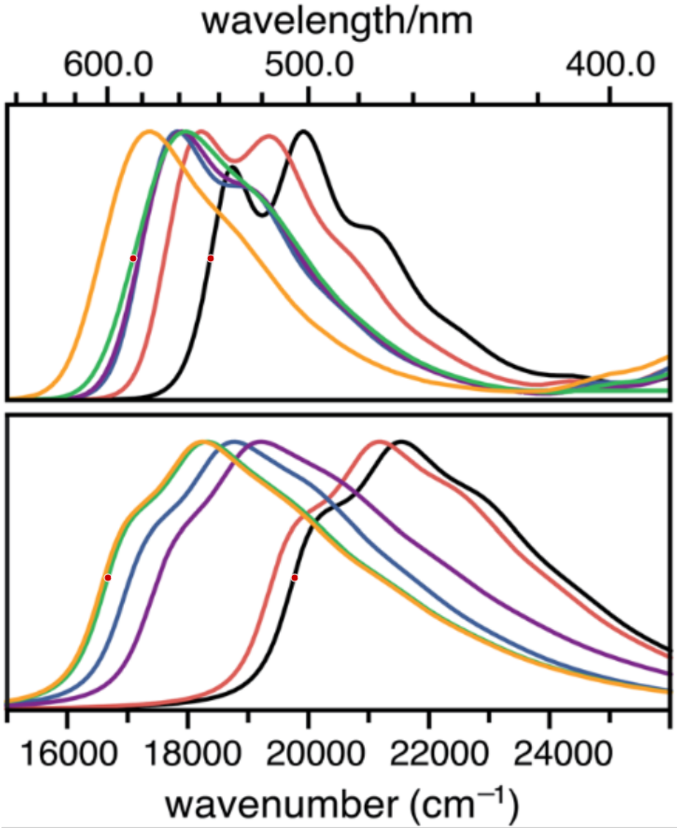

Figure S4: Digitized experimental absorption spectra (top) and calculated absorption spectra at the TDDFT/IEFPCM (def2-TZVP) level (bottom) from fig. 2 of ref. 17 (reproduced with permission, copyright 2019 Royal Society of Chemistry) for DCBT in various solvents. The spectra in green refer to DCBT in acetonitrile (ACN) and the ones in black refer to DCBT in methylcyclohexane (MCH). Red points indicate the left inflection point of each relevant band which are selected to calculate the solvatochromic shift to reduce the error arising from the neglect of nuclear structure effects in the transition.

Table S8: Wavenumber ($\tilde{\nu}$) and corresponding frequencies (ω) selected in figure S4 to calculate the solvatochromic shifts (δ) between acetonitrile (ACN) and methylcyclohexane (MCH) in the $\pi \rightarrow \pi^*$ transition of DCBT. The wavenumber values are extracted using the WebPlotDigitizer software[19] and are converted to frequencies.

	$\tilde{\nu}_{MCH}(cm^{-1})$	$\tilde{\nu}_{ACN}(cm^{-1})$	$\omega_{MCH}(eV)$	$\omega_{ACN}(eV)$	$\delta\ (eV)$
experimental	17084	18307	2.12	2.27	-0.15
TDDFT/IEFPCM (def2-TZVP)	16666	19764	2.07	2.45	-0.38

Table S9: Frequencies (ω) selected in fig. 2 of ref. 18 to calculate the solvatochromic shift (δ) between dimethylsulfoxide (DMSO) and gas-phase and the solvatochromic shift (Δ) between toluene (TOL) and gas-phase in the $\pi \rightarrow \pi^*$ transition of DCBT. The frequency values are extracted using the WebPlotDigitizer software.[19]

	$\omega_{gas-phase}(eV)$	$\omega_{DMSO}(eV)$	$\delta\ (eV)$	$\omega_{TOL}(eV)$	$\Delta\ (eV)$
SA(2)-CASSCF/C-PCM (6,5)	4.44	3.68	-0.76	4.04	-0.40

References

(1) Abraham, M. J.; Murtola, T.; Schulz, R.; Páll, S.; Smith, J. C.; Hess, B.; Lindahl, E. GROMACS: High performance molecular simulations through multi-level parallelism from laptops to supercomputers. *SoftwareX* **2015**, *1*, 19–25.

(2) Sousa da Silva, A. W.; Vranken, W. F. ACPYPE - AnteChamber PYthon Parser interfacE. *BMC Research Notes* **2012**, *5*, 367.

(3) Wang, J.; Wang, W.; Kollman, P. A.; Case, D. A. Automatic atom type and bond type perception in molecular mechanical calculations. *Journal of Molecular Graphics and Modelling* **2006**, *25*, 247–260.

(4) Wang, J.; Wolf, R. M.; Caldwell, J. W.; Kollman, P. A.; Case, D. A. Development and testing of a general amber force field. *J. Comput. Chem.* **2004**, *25*, 1157–1174.

(5) Jakalian, A.; Jack, D. B.; Bayly, C. I. Fast, efficient generation of high-quality atomic charges. AM1-BCC model: II. Parameterization and validation. *Journal of Computational Chemistry* **2002**, *23*, 1623–1641.

(6) Frisch, M. J. et al. Gaussian 16 Revision C.01. 2016; Gaussian Inc. Wallingford CT.

(7) Head-Gordon, M.; Pople, J. A.; Frisch, M. J. MP2 energy evaluation by direct methods. *Chemical Physics Letters* **1988**, *153*, 503–506.

(8) Head-Gordon, M.; Head-Gordon, T. Analytic MP2 frequencies without fifth-order storage. Theory and application to bifurcated hydrogen bonds in the water hexamer. *Chemical Physics Letters* **1994**, *220*, 122–128.

(9) Lipparini, F.; Scalmani, G.; Mennucci, B.; Cancès, E.; Caricato, M.; Frisch, M. J. A variational formulation of the polarizable continuum model. *The Journal of Chemical Physics* **2010**, *133*, 014106.

(10) Kowsari, M. H.; Tohidifar, L. Systematic evaluation and refinement of existing all-atom force fields for the simulation of liquid acetonitrile. *J. Comput. Chem.* **2018**, *39*, 1843–1853.

(11) Darden, T.; York, D.; Pedersen, L. Particle mesh Ewald: An $N \cdot \log(N)$ method for Ewald sums in large system. *The Journal of Chemical Physics* **1993**, *98*, 10089–10092.

(12) Hess, B.; Bekker, H.; Berendsen, H. J. C.; Fraaije, J. G. E. M. LINCS: A linear constraint solver for molecular simulations. *Journal of Computational Chemistry* **1998**, *18*, 1463–1472.

(13) Hess, B. P-LINCS: A Parallel Linear Constraint Solver for Molecular Simulation. *Journal of Chemistry Theory and Computation* **2008**, *4*, 116–122.

(14) Michaud-Agrawal, N.; Denning, E. J.; Woolf, T. B.; Beckstein, O. MDAnalysis: a toolkit for the analysis of molecular dynamics simulations. *Journal of Computational Chemistry* **2011**, *32*, 2319–2327.

(15) Renge, I. Solvent Dependence of n- π* Absorption in Acetone. *J. Phys. Chem. A* **2009**, *113*, 10678–10686.

(16) Bayliss, N.; Wills-Johnson, G. Solvent effects on the intensities of the weak ultraviolet spectra of ketones and nitroparaffins—I. *Spectrochim. Acta A* **1968**, *24*, 551–561.

(17) Hoche, J.; Schulz, A.; Dietrich, L. M.; Humeniuk, A.; Stolte, M.; Schmidt, D.; Brixner, T.; Würthner, F.; Mitric, R. The origin of the solvent dependence of fluorescence quantum yields in dipolar merocyanine dyes. *Chem. Sci.* **2019**, *10*, 11013–11022.

(18) Song, C. State-averaged CASSCF with polarizable continuum model for studying photoreactions in solvents: Energies, analytical nuclear gradients, and non-adiabatic couplings. *J. Chem. Phys.* **2022**, *156*, 104102.

(19) Rohatgi, A. WebPlotDigitizer. `https://automeris.io`.
